# Achieving Persistent Luminescence Performance Based on the Cation-Tunable Trap Distribution

**DOI:** 10.3390/ma15249083

**Published:** 2022-12-19

**Authors:** Tao Wang, Rui Li, Mengya Zhang, Panlai Li, Zhijun Wang

**Affiliations:** 1 College of Science, China University of Petroleum (East China), Qingdao 266580, China; 2 Hebei Key Laboratory of Optic-Electronic Information and Materials, College of Physics Science & Technology, Hebei University, Baoding 071002, China

**Keywords:** phosphor, persistent luminescence, trap regulation, tunneling channel

## Abstract

Deep-red persistent luminescence (PersL) materials have promising applications in fluorescence labeling and tracking. PersL spectral range and PersL duration are considered to be the key factors driving the development of high-performance deep-red PersL materials. To address these two key issues, the performance of PersL materials was continually optimized by doping with cations (Si^4+^ and Al^3+^ ions), relying on the material of Li_2_ZnGe_3_O_8_:Cr^3+^ from the previous work of our group, and a 4.8-fold increase in PersL radiation spectrum intensity and more than twice the PersL duration was achieved (PersL duration up to 47 h). Ultimately, the obtained PersL materials are used to demonstrate their potential use in multi-level anti-counterfeiting, tracking and localization, respectively. This study provides a unique and novel entry point for achieving high-performance PersL materials by optimizing the PersL material host to modulate the electronic structure.

## 1. Introduction

PersL materials have recently attracted great enthusiasm among researchers due to their low background interference and high spatial resolution and are increasingly being used in many applications, such as anti-counterfeiting and bio-penetration [1,2,3,4,5,6,7,8,9,10]. Currently, a whole host of fluorescence is increasingly used for in vivo imaging and provides remarkable results. However, this technique has several limitations, especially due to tissue autofluorescence under external illumination and weak tissue penetration with low-wavelength excitation light. However, for PersL as a unique property, self-sustained radiation can persist for seconds to hours, even after external excitation is stopped, so longer-wavelength PersL materials are gradually demonstrating their advantages for biofluorescence imaging [11,12,13,14,15,16,17]. In recent years, although a large number of PersL materials have been developed that can be effectively charged by UV-red light, they are difficult to satisfy at longer wavelengths and tend to be just short in duration, for example, CaAl_2_O_4_:Eu^2+^, Nd^3+^ (blue) [18], SrAl_2_O_4_:Eu^2+^, Dy^3+^ (green) [19] and Y_2_O_2_S: Eu^3+^, Mg^2+^, Ti^4+^ (red) [20], Ca_0.2_Zn_0.9_Mg_0.9_Si_2_O_6_:Eu^2+^, Dy^3+^, Mn^2+^ (NIR) [16], hence, excellent PersL materials are still a meaningful and challenging work.

In addition, the research on the emission mechanism of PersL is still imperfect, which limits the development of PersL materials. In fact, the basic principle of PersL materials is related to two kinds of active centers: emission center and trap center. The former allows the material to emit light in the wavelength range of interest and the latter helps to prolong the continuous luminescence time of PersL materials. Aiming at the problem of insufficient properties of PersL materials, how to slow down the attenuation rate of PersL has become one of the problems to be solved immediately. For the goal of optimizing the PersL properties of materials, researchers have adopted different methods: (1) adjusting the PersL duration by introducing metal cations to optimize the lattice occupation [21,22,23,24]; (2) extending the PersL emission time by using a PersL emission mechanism (such as using general bandgap engineering) [25]; (3) introducing new trap energy levels by co-doping cations or rare-earth ions with different valence states to delay the decay rate of PersL [26,27,28,29,30]; (4) abandoning the traditional X-ray or ultraviolet (UV) excitation and using visible light to excite a few suitable materials to achieve the ideal state [31].

In our previous work, by adjusting the doping concentration of Cr^3+^ ions, LZG exhibited a bright-red PersL emission with a peak of 698 nm for 20 h. The analysis of PersL decay curves and TL spectra of a series of samples reveals that the PersL luminescence mechanism of LZG:Cr involves two channel models (conduction band (CB) channel and tunneling (TN) channel) through which carriers pass [32]. The PersL emission generated by the carrier of the CB channel is very bright but short, while the PersL generated by the carrier dominated by the TN channel is slow but relatively dark. The greater the concentration of shallow traps, the more carriers there are in the CB channel in the material. Conversely, the larger the concentration of deep traps, the more carriers in the TN channel in the material. Therefore, the relationship between the ratio of deep and shallow traps in the material has a great influence on the performance of PersL emission [33].

Here, Li_2_ZnGe_3_O_8_:Cr^3+^ modulated by cations (Si^4+^ ions and Al^3+^ ions) with efficient UV charging ability PersL material was studied by us. The ratio of deep and shallow traps was optimized by adjusting the host composition with Si^4+^ ions and Al^3+^ ions, thus, changing the number of carriers in the CB and TN channels and finally improving the emission performance of PersL with a 4.8-fold increase in the intensity of PersL spectrum and a PersL duration of 47 h. By means of X-ray diffraction spectroscopy, TL spectroscopy and transient fluorescence spectroscopy, the detailed PersL emission mechanism and material defect distribution were explored, which not only confirmed the correctness of the strategy proposed in this work, but also provided an entry point for obtaining new excellent PersL materials. In the end, its wide potential applications are revealed through its application in the fields of secret labeling and dynamic anti-counterfeiting.

## 2. Materials and Methods

Li_2_ZnGe_3_O_8_: Cr^3+^ (LZG: Cr^3+^), Li_2_ZnGe_3-y_Si_y_O_8_: Cr^3+^ (LZGS: Cr^3+^) and Li_2_Zn_1-z_Ge_3-z_Al_2z_O_8_: Cr^3+^ (LZGA: Cr^3+^) were synthesized via the high-temperature solid-state method. The stoichiometric amount of Li_2_CO_3_ (A.R.), ZnO (A.R.), SiO_2_ (99.99%), GeO_2_ (99.99%), Al_2_O_3_ (99.99%) and Cr_2_O_3_ (99.99%) was mixed in an agate mortar for 30 min. Then, the mixed powder was transformed into an alumina crucible and kept in a high-temperature furnace at 975 °C for 3 h. Then, the sample was naturally cooled and after reaching room temperature, the sample was again ground to obtain a finely powdered sample.

The phase formation of samples was examined by X-ray powder diffraction (XRD) performed on a Bruker D8 X-ray diffractometer (Bruker, München, Germany) with Ni-filtered Cu Kα radiation (λ = 0.15405 nm), operating at 40 mA, 40 kV with step length and diffraction range was 0.05° and 10° to 80°, respectively. Crystal structure refinements employing the Rietveld method were implemented using the General Structure Analysis System (GSAS) software (version 1251). Room-temperature photoluminescence spectra of samples were recorded with a Hitachi F-4600 fluorescence spectrophotometer using a 450 W Xe lamp as the excitation source, with a scanning wavelength from 200 to 900 nm, scanning at 240 nm/min. The reflect spectra were measured by HITACHI U4100 (HITACHI, Tokyo, Japan) in a range of 200 to 700 nm. The TL curves were recorded by an FJ-427A1 TL dosimeter with a fixed heating rate of 1 °C/s within a range of 300–600 K. At room temperature, Horiba FL3 was used to measure the life decay curve and PersL decay curve of the phosphor powder. The width of the entrance and exit slit of the test life decay curve was 0.5 nm, and the width of the PersL curve of the test sample was 29 nm. The microstructure and morphology of powder crystals were measured by field-emission scanning electron microscopy (SEM, Nova Nano 450). The elemental composition and distribution were determined using an energy dispersive X-ray spectroscope attached to SEM.

## 3. Results and Discussion

### 3.1. Optimization of PersL Decay Rate by Doping Si^4+^ Ions

#### 3.1.1. Phase Information of Li_2_ZnGe_3-y_Si_y_O_8_: 0.8%Cr^3+^

**Figure 1:**
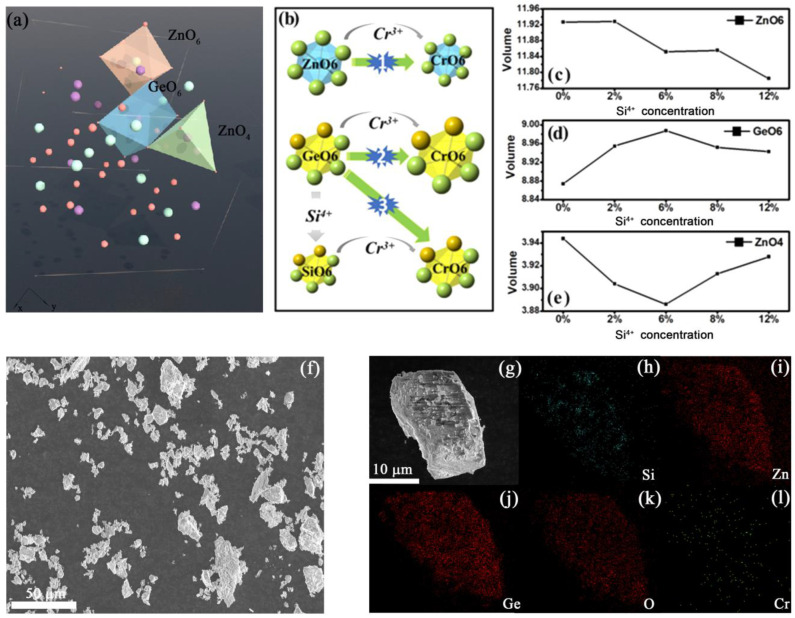
(**a**) Schematic of the crystal structure of LZG; (**b**) simulation diagram of variation trend of each cell volume with the increase in y value in Li_2_ZnGe_3-y_Si_y_O_8_: 0.8%Cr^3+^; the volume of (**c**) ZnO6 (**d**) GeO6 and (**e**) ZnO4 unit cell in Li_2_ZnGe_3-y_Si_y_O_8_: 0.8%Cr^3+^ with increasing y, respectively; (**f**) SEM and (**g**–**l**) elemental mapping analysis of Li_2_ZnGe_3-y_Si_y_O_8_: 0.8%Cr^3+^.

LZG is known to have a spinel structure, which is cubic with the space group *P4_3_32*. In this case, Li_1_/Zn_1_ occupies the tetrahedral sites while the other Li_2_/Zn_2_ and all of the Ge occupy the octahedral sites (Figure 1a), and Cr^3+^ ions can only enter the 6-coordinated sites (ZnO6 and GeO6). The XRD patterns of LZGS:Cr are shown in Figure S1 (see Supplementary Materials). All of the diffraction peaks match well with PDF #24-0673. Figure S2 and Table S1 (see Supplementary Materials) depict the Rietveld refinement XRD pattern of LZG and LZGS: Cr. The low *R* factors indicated that the structural refinement is reliable (R_wp_ = 10.57%, R_p_ = 8.63%, χ^2^ = 1.450). After matrix regulation, there are three suitable positions (ZnO6, GeO6 and SiO6) for Cr^3+^ ions to enter (Figure 1b). Because the radius of Cr^3+^ ions (r = 0.615 Å, CN = 6) is smaller than that of Zn^2+^ ions (r = 0.74 Å, CN = 6), the volume of ZnO6 cells decreases (process 1). Similarly, when Cr^3+^ ions occupy Ge^4+^ ions (r = 0.53 Å, CN = 6), the volume of GeO6 cells increases (process 2). In addition, it is worth noting that when Cr^3+^ ions occupy Si^4+^ ions (r = 0.40 Å, CN = 6), two processes (process 3) affecting the cell volume are involved: 1) Si^4+^ ions successfully enter the GeO6 position of LZG and the cell volume decreases. 2) Cr^3+^ ions successfully occupy SiO6 sites and the cell volume increases. The detailed crystal lattice occupancy can be explained by the refined plot. When the matrix is regulated by Si^4+^ ions, the cell volume at the ZnO6 site decreases (Figure 1c), indicating that Cr^3+^ ions occupying this site increase. The cell volume of the GeO6 site first increases and then decreases (Figure 1d), with the low Si^4+^ ion concentration; the possibility of process 2 is greater than that of process 3. With the increasing concentration of Si^4+^ ions, the number of SiO6 and the effect of lattice distortion gradually increase, resulting in the possibility of process 3 being greater than that of process 2. Most of the oxygen atoms in ZnO4 are shared with GeO6, hence, the change in GeO6 volume will inevitably pull or squeeze ZnO4, as shown in Figure 1e (the change in ZnO4 volume is inversely proportional to GeO6). To sum up, with the increase in Si^4+^ ion concentration, Cr^3+^ ions occupy more ZnO6 and SiO6 and less GeO6. The average particle size of the synthesized LZGS: Cr microcrystal particles was 50 μm according to the morphological observation (Figure 1f). Energy dispersive spectroscopy elemental mapping analysis showed nearly homogeneous elemental distributions (Figure 1g–l). These results also demonstrate excellent crystallinity in the material.

#### 3.1.2. PersL Properties of Li_2_ZnGe_3-y_Si_y_O_8_: 0.8%Cr^3+^

**Figure 2:**
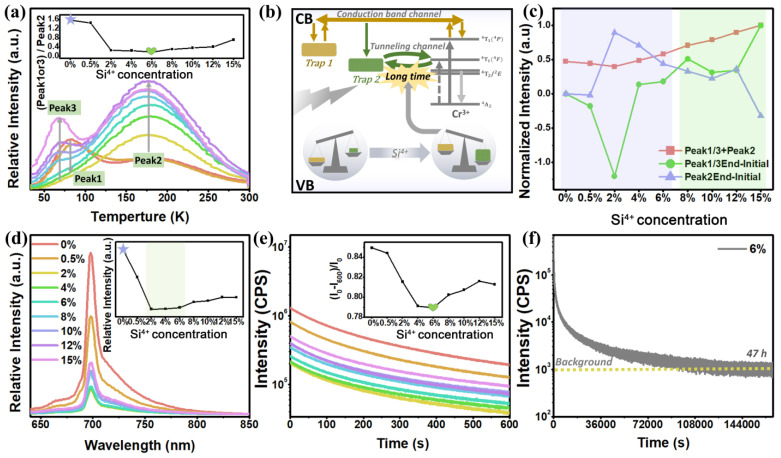
(**a**) The TL spectra of Li_2_ZnGe_3-y_Si_y_O_8_: 0.8%Cr^3+^ (*y* = 0–15%); inset: the proportional relationship between the intensity of TL spectra of shallow trap (peak 1 or 3) and deep trap (peak 2); (**b**) mechanism of PersL emission on CB channel and TN channel; (**c**) analytical plot of the effect of TL spectral trap distribution on the PersL emission performance (emission intensity and attenuation time) of Li_2_ZnGe_3-y_Si_y_O_8_: 0.8%Cr^3+^; (**d**) long PersL spectra of L Li_2_ZnGe_3-y_Si_y_O_8_: 0.8%Cr^3+^; (**e**) PersL decay curve of Li_2_ZnGe_3-y_Si_y_O_8_: 0.8%Cr^3+^; inset: trend of decay rate of PersL at different y values; (**f**) the PersL attenuation curve of Li_2_ZnGe_3-y_Si_y_O_8_: 0.8%Cr^3+^ (*y* = 6%).

The prepared samples can also exhibit a good red PersL after being exposed to UV irradiation. Figure 2a demonstrates that LZGS:0.8%Cr^3+^ contains a shallow trap (peak 1 and peak 3) and a deep trap (peak 2). With the continuous doping of Si^4+^ ions, the deep trap continues to increase, the shallow trap first decreases and then increases and the position of the shallow trap moves from “deep” to “shallow”. According to the trap depth equation:

(1) $$E=\frac{T_{m}}{500}$$

the trap depth can be calculated as 0.904 eV (peak 2).

When Zn^2+^ ions are replaced by Cr^3+^ ions, due to the unequal substitution of charges, defects will be generated to balance the internal charges:

(2) $${2\mathrm{Cr}}^{3+}{+2\mathrm{Zn}}^{2+}\;\to {\;2\mathrm{Cr}}_{\mathrm{Zn}}^{\;.}{+O}_{i}^{\prime \prime }$$

Similarly, when Ge^4+^ and Si^4+^ ions are replaced by Cr^3+^, defects will also occur:

(3) $${2\mathrm{Cr}}^{3+}{+2\mathrm{Ge}}^{4+}\;\to {\;2\mathrm{Cr}}_{\mathrm{Ge}}^{\;\prime }{+V}_{\overset{..}{O}}$$

(4) $${2\mathrm{Cr}}^{3+}{+2\mathrm{Si}}^{4+}\;\to {\;2\mathrm{Cr}}_{\mathrm{Si}}^{\;\prime }{+V}_{\overset{..}{O}}$$

When the doping concentration of Si^4+^ ions reaches a certain value, the internal charge has been balanced and there will be no new defects:

(5) $${4\mathrm{Cr}}^{3+}\;\to {\;2\mathrm{Zn}}^{2+}{+\mathrm{Ge}}^{4+}{+\mathrm{Si}}^{4+}\;$$

The deep trap is the electronic defect of ${\mathrm{Cr}}_{\mathrm{Zn}}^{\;.}$ and the shallow trap is electronic defect of $V_{\overset{..}{O}}$ [32]. When the incorporation of Si^4+^ ions is increased, the intensity of the shallow trap first decreases and then increases, while the trap position gradually moves from peak 1 to peak 3, which may be caused by internal structural changes. When 0% < y < 2%, crystallographic lattice of Cr^3+^ occupying the ZnO6 crystallographic lattice leads to the continuous increase in peak 2 (process (1)), the content of Si^4+^ ions in the material is reduced and the number of electrons eliminated in process (4) is gsreater than that of electrons increased in process (2); hence, peak 1 continues to decline until it disappears. When the concentration of Si^4+^ ions increases to 2% < y < 12%, the possibility of the crystallographic site of SiO6 being occupied by the crystallographic site of Cr^3+^ increased, which leads to the appearance of peak 3 and a continuous increase in the intensity of TL spectra (process (3)).

The concentration of deep traps is promoted by modulation with Si^4+^ and the TN channel has more carriers, and there is an ultra-long but dark PersL emission (Figure 2b). The band gap value of 5.04 eV was found by diffuse reflectance spectra (DRs) (Figure S3). The proportion of deep and shallow traps may be expressed by the following formula, as shown in the inset in Figure 2c:

[peak 1 or 3]/[peak 2](6)

among them, [peak 1 or 3] is a shallow trap and peak 2 is a deep trap. A larger ratio of [peak 1 or 3]/[peak 2] means that the intensity of PersL emission is very high and dominated by the CB channel, while a smaller ratio means that the PersL attenuation time is dominated for a very long time by the TN channel [34]. When combined with Figure 2d,e, it can be inferred that, at *y* = 6%, the PersL attenuation duration of the material reaches the longest, and at *y* = 0%, the PersL emission intensity of the material is the highest. The PersL spectra (Figure 2d) and PersL attenuation curve (Figure 2e) of samples verify the correctness of the conclusion. By doping Si^4+^ ions (LZGS: Cr^3+^), the trap ratio is successfully adjusted and the attenuation rate of PersL is optimized, so that the emission time of PersL reaches 47 h (Figure 2f), which is more than twice as long as before (LZG: Cr^3+^).

The trends in the PersL emission intensity (inset in Figure 2d) and decay rate (inset in Figure 2e) remain consistent, but there are still slight differences (y = 2–6%), which can be explained by Figure 2c. The PersL emission intensity is determined by the number of carriers in the trap, that is, the intensity of TL. When the material contains multiple traps, the sum of the TL intensities of different traps ((peak 1or 3) + (peak 2)) can be simply regarded as the overall PersL emission intensity. The value of ((peak 1or 3) + (peak 2)) first decreased and then increased and reached the lowest at y = 2% (red line in Figure 2c), which is in perfect agreement with the variation in PersL emission intensity in Figure 2d. Since the decay rate of PersL is determined by the simultaneous processing of two channels (CB and TN channel), what needs to be judged is which channel plays a relatively decisive role. When the y value changes, the variation amplitude of the two traps ((Peak 1 or 3) End-Initial and (Peak 2) End-Initial) affects which carrier channel plays an absolute role. As can be seen from Figure 2c, when y = 0–6%, the blue line is above the green line, so the dominant TN channel slows down the decay rate of the PersL, resulting in a longer PersL emission time. When y = 8–15%, the green line is above the blue line, that is, the dominant CB channel will speed up the emission attenuation speed of the PersL. The above analysis is consistent with the variation trend of the PersL attenuation speed (Figure 2e) and it fully validates our conclusion.

### 3.2. Optimization of PersL Emission Intensity by Doping Al^3+^ Ions

#### 3.2.1. Phase Information of Li_2_Zn_1-z_Ge_3-z_Al_2z_O_8_: 0.4%Cr^3+^

**Figure 3:**
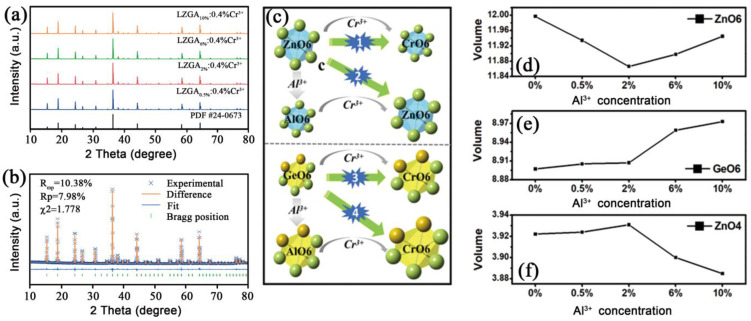
(**a**) XRD patterns of all samples; **(b**) the Rietveld structure refinement of Li_2_Zn_1-z_Ge_3-z_Al_2z_O_8_: 0.4%Cr^3+^; (**c**) simulation diagram of variation trend of each cell volume with the increase in *z* value in Li_2_Zn_1-z_Ge_3-z_Al_2z_O_8_: 0.4%Cr^3+^; the volume of (**d**) ZnO6 (**e**) GeO6 and (**f**) ZnO4 unit cell in Li_2_Zn_1-z_Ge_3-z_Al_2z_O_8_: 0.4%Cr^3+^ with increasing *z*, respectively.

As shown in Figure 3a, the XRD patterns of all samples present that no impurity peaks were observed compared to the standard card of LZG (PDF#24-0673), indicating that the synthesized samples were pure LZGA: Cr phase. Furthermore, to investigate the crystal structure of LZGA: Cr, the Rietveld structure refinement of LZGA: Cr was studied using GSAS software, as depicted in Figure 3b and Table S2. The reliability coefficients of the refinement are R_wp_ = 10.38% and R_p_ = 7.98%, which indicate that the results are reliable. After the host is regulated by Al^3+^ ions, there are four suitable positions for Cr^3+^ ions to enter, as shown in Figure 3c, namely ZnO6, GeO6, AlO6[Ge] and AlO6[Zn]. Compared with Zn^2+^, the valence state of Cr^3+^ ions is closer to that of Al^3+^ ions. Therefore, with the increase in Al^3+^ ions, process 2 gradually becomes dominant. Under the combined action of processes 1 and 2, the cell volume of ZnO6 first decreases and then increases (Figure 3d). The cell volume of GeO6 (Figure 3e) is the joint action of process 3 and 4, which also proves that Cr^3+^ ions are successfully incorporated into GeO6 and AlO6[Ge]. Since the radius of Al^3+^ ion (r = 0.535 Å, CN = 6) is closer to that of Ge^4+^ ion (r = 0.53 Å, CN = 6), we believe that the greater the z value, the greater the probability of process 4 [32]. Similarly, the volume of ZnO4 (Figure 3f) is inversely proportional to that of GeO6, which is due to the sharing of oxygen atoms. To sum up, with the increase in Al^3+^ ion concentration, the possibility of occupation of ZnO6 and GeO6 by Cr^3+^ ions decreases, and the possibility of occupying AlO6[Ge] is greater than AlO6[Zn]. In addition, the SEM revealed the regular shape and smooth surface of the samples (Figure S4). The elemental mapping analysis images demonstrated the uniform distribution of the constituent elements (Zn, Ge, Al, O and Cr) within a single particle.

#### 3.2.2. PersL Properties of Li_2_Zn_1-z_Ge_3-z_Al_2z_O_8_: 0.4%Cr^3+^

**Figure 4:**
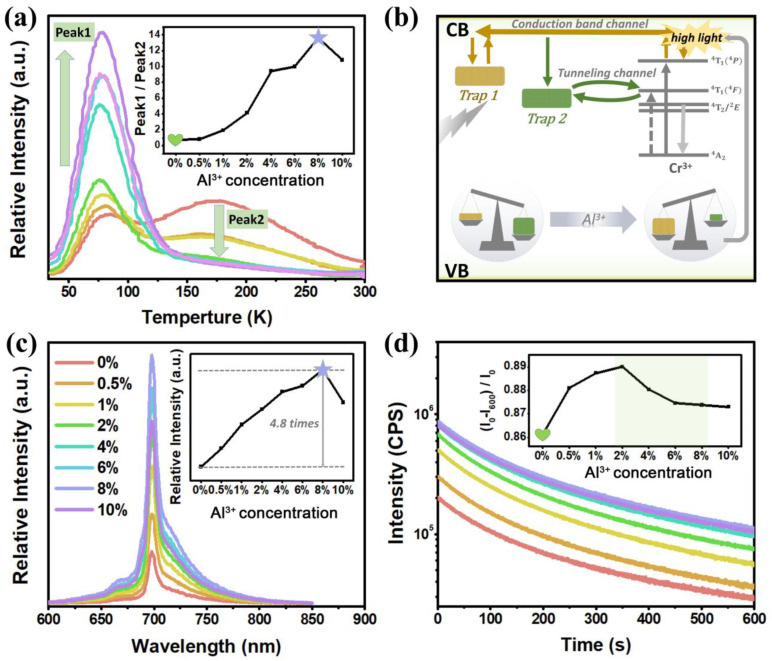
Properties of LPL. (**a**) TL spectra of Li_2_Zn_1-z_Ge_3-z_Al_2z_O_8_: 0.4%Cr^3+^ (z = 0–10%), inset: the proportional relationship between the TL intensity of shallow trap (peak 1) and deep trap (peak 2); (**b**) effect plot of CB channel and TN channel on PersL emission; (**c**) PersL spectra of Li_2_Zn_1-z_Ge_3-z_Al_2z_O_8_: 0.4%Cr^3+^; (**d**) PersL decay curve of Li_2_Zn_1-z_Ge_3-z_Al_2z_O_8_: 0.4%Cr^3+^, inset: trend of decay rate of PersL at different z values.

From the TL spectra of LZGA:0.4%Cr3+ (Figure 4a), it can be seen that the trap position of the material is the same as before, including a deep trap and a shallow trap. With the increase in Al3+ ions, the concentration of the shallow trap (peak 1) increases and the concentration of the deep trap (peak 2) decreases. According to Equation (1), the trap depth can be calculated as 0.706 eV (peak 1).

When the ZnO6 site is replaced by Cr^3+^ ions, defects will be generated to balance the internal charge due to unequal substitution. When the AlO6[Zn] site is replaced by the Cr^3+^ site, since Al^3+^ ions first replace Zn^2+^ ions, and then Cr^3+^ ions replace Al^3+^ ions occupying this site, it is equivalent that Al^3+^ ions act as an "intermediary". The defect generation processes of both are as in Equation (2). Similarly, when GeO6 and AlO6[Ge] are replaced by Cr^3+^, the defect generation process is as in Equation (3). As Al^3+^ ions are doped, the charge in the material reaches a certain balance value and the internal charge gradually tends to balance:

(7) $${4\mathrm{Cr}}^{3+}\to {\mathrm{Zn}}^{2+}{+\mathrm{Ge}}^{4+}{+2\mathrm{Al}}^{3+}\;$$

It is determined that peak 1 is still $V_{\overset{..}{O}}$ defect, peak2 is still $Cr_{Zn}^{\;.}$ defect [32,35,36,37]. As the concentration of Al^3+^ ions increases, the occupation of ZnO6 and GeO6 by Cr^3+^ ions decreases, but with the increased occupation of AlO6, the possibility of occupying AlO6[Ge] is greater than AlO6[Zn]. When Al^3+^ ion is doped with a low concentration (y = 0–8%), $Cr_{Zn}^{\;.}$ defects in the material decrease (process (6)) and $V_{\overset{..}{O}}$ defects increase (process (7)), which led to the rise in peak 1 and the decline in peak 2 in this range. When the Al^3+^ ion concentration continues to increase, peak 1 decreases slightly, which is due to the increase in the process (8) offset effect.

By adding Al^3+^ ions into the matrix, the proportion of deep and shallow trap concentration in the material changed significantly, which is analyzed by the following formula:

peak 1/peak 2(8)

Among them, peak 1 represents a shallow trap and peak 2 represents a deep trap. A larger ratio of peak 1/peak 2 means that there are more shallow traps, hence, the dominant CB channel leads to bright PersL emission (Figure 4b). It can be seen from the inset in Figure 4a that the ratio of peak 1/peak 2 first increases and then decreases slightly. It can be inferred that when z = 8%, the PersL emission intensity of the sample is the highest, while when z = 0%, the PersL attenuation speed of the material is the slowest. Figure 4c depicts the PersL emission spectra of LZGA:0.4%Cr^3+^, and the intensity of PersL emission reaches the maximum at z = 8%, which is 4.8-times higher than that of the previous (LZG: Cr^3+^). The inset in Figure 4d shows the PersL decay rate of samples. It can be seen that, at z = 0%, the PersL decay rate is indeed the slowest, and then with the increase in z value, the PersL decay rate increases first and then slows down. When 0% < z < 2%, the intensity of peak 1 is increased and peak 2 is decreased, and the dominant shallow trap leads to a faster attenuation rate. When 2% < z < 10%, the intensity of peak 1 is also high at this time; although the attenuation speed is faster, it still takes some time to fully attenuate in a short time (600 s). It can also be explained that assuming 100 electrons, the original attenuation rate is 10 and the attenuation time is 10. When the concentration increases to 500, the attenuation rate increases to 25, but the attenuation time is 20, which is longer than the previous 10. 

### 3.3. Applications

**Figure 5:**
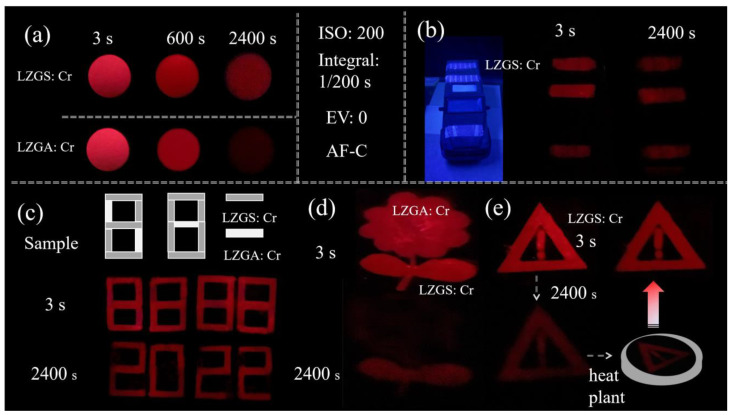
(**a**,**b**) Digital photos of designed dynamic color images at different times; (**c**–**e**) encryption application of the luminescence image in the virtual scenario.

According to the unique characteristics of LZGS: Cr and LZGA: Cr phosphors, the luminescence images were recorded for secret labeling and dynamic anti-counterfeiting, as shown in Figure 5. Our camera used the following series of parameters: ISO:200, Integral:1/200 s, EV: 0, AF-C, and WB-AWB, using the same settings when taking digital photographs to ensure the authenticity and accuracy of the image data. Figure 5a displays the PersL images of LZGS: Cr and LZGA: Cr at different times after UV irradiation for 15 min. It can be clearly seen that the PersL luminescence of LZGA: Cr at 3 s is brighter than that of LZGS: Cr. However, the PersL of LZGA: Cr almost disappears after 2400 s, but the brightness of LZGS: Cr can still be detected; hence, we use the material’s PersL as a secret light source for tracking and localization (Figure 5b). We applied LZGS: Cr to the surface of the model car, and after 5 min of 254 nm UV lamp excitation, the stripes glowed in the dark due to the self-sustained dark-red PersL emission, and after 2400 s, the dark-red stripes could still be clearly monitored by an ordinary camera. Thus, LZGS: Cr is a potentially efficient coating additive that can be used for marking friendly or unfriendly devices.

In addition, some information is hidden by persistent luminescence used to design the anti-counterfeit label. Figure 5c–e display the digital photographs of the three designed images. It is found that the designed images show different hidden information at different times after stopping UV irradiation. The letter ‘8888’ changes to ‘2022’ at 3 s and 3000 s after stopping UV irradiation in Figure 5c. The sample of LZGA: Cr with fast decay rate was filled with the flower and the sample of LZGS: Cr with slow decay rate was filled with the position of leaf, and after 2400 s, the flower almost disappeared (as in Figure 5d). 

Finally, we designed anti-counterfeiting by using trap energy storage, as heating the sample can excite the PersL emission. The warning label in Figure 5e decreases in brightness from 3 s to 2400 s, and the sample after decaying through 2400 s placed on a heating plate at a temperature of 423 K can appear as a bright warning label again. It is shown that the advantage of trap heating can be used to release electrons again and design dynamic anti-counterfeiting.

## 4. Conclusions

In summary, we optimized the proportion of deep and shallow traps by adjusting the matrix composition by Si^4+^ ions and Al^3+^ ions, so as to change the carrier amount of CB channel and TN channel, and finally improve the emission performance of PersL. The decay time of PersL is more than 2-times (up to 47 h) and the emission intensity of PersL is more than 4.8-fold. In the end, its wide range of potential applications is revealed through its application in the field of secret label and dynamic anti-counterfeiting. This work provides constructive ideas for rational optimization of material properties, preparation of high-performance PersL materials and the development of new advanced applications.

## Data Availability

Not applicable.

## Supplementary Materials

The following supporting information can be downloaded at: https://www.mdpi.com/article/10.3390/ma15249083/s1, Figure S1: The XRD of Li_2_ZnGe_3-y_Si_y_O_8_: 0.8%Cr^3+^ (x = 0–12%); Figure S2: The Rietveld refinement results of (a) LZGO and (b) LZGS_6%_O:0.8%Cr^3+^; Figure S3: The DRs of LZGA: Cr, LZGS: Cr and LZGO: Cr. Inset: the bandgap width of LZGA: Cr, LZGS: Cr and LZGO: Cr; Figure S4: Elemental mapping analysis and SEM of LZGAO:0.8%Cr^3+^; Table S1: Refined crystallographic data of LZGS: Cr (y = 0–12%); Table S2: Refined crystallographic data of LZGA: Cr (y = 0–12%).
