# Supplementary material for: Achieving Persistent Luminescence Performance Based on the Cation-Tunable Trap Distribution
Source: Materials (Basel). 2022 Dec 19;15(24):9083. doi: 10.3390/ma15249083 (PMC9782221; doi:10.3390/ma15249083)
Supplement: Supplementary file 1 [file materials-15-09083-s001.zip › materials-2079149-supplementary.pdf]

Supplementary materials

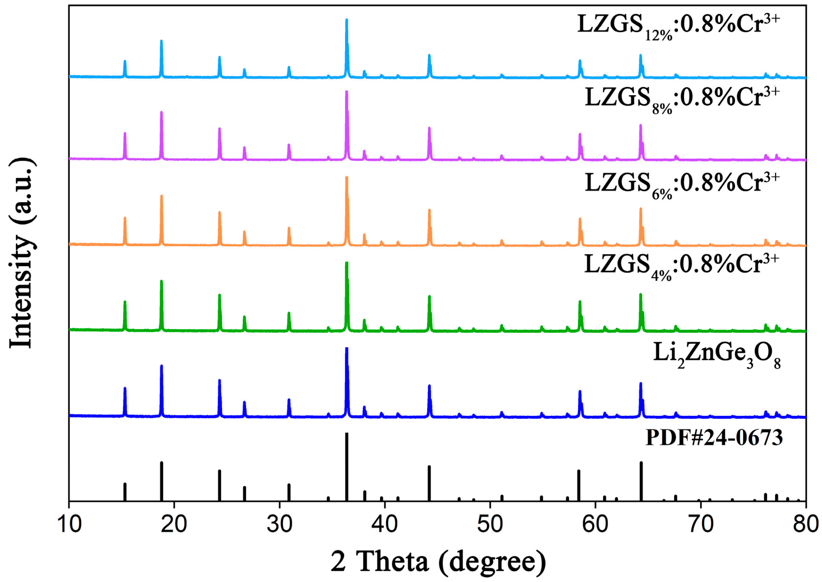

Figure S1. The XRD of LZGSxO:0.8%Cr^{3+} (x = 0-12%).

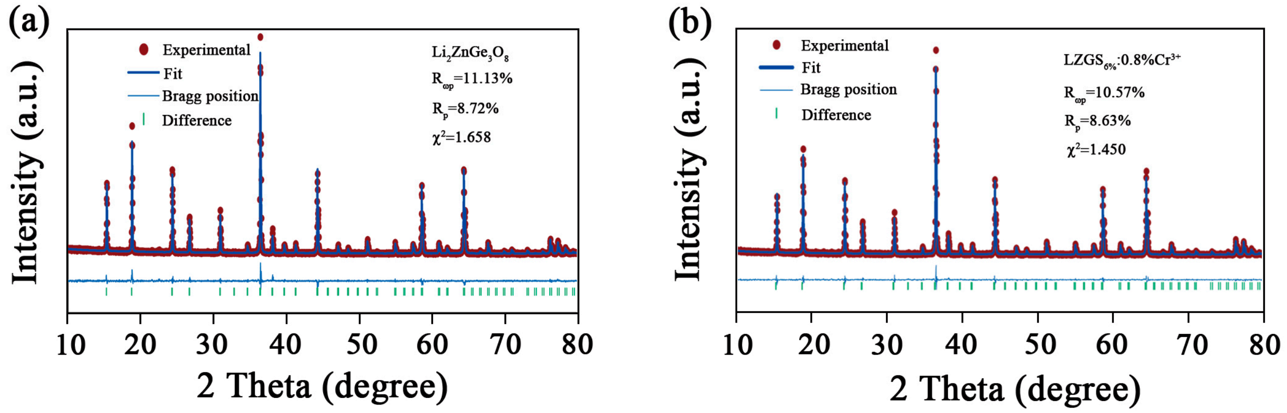

Figure S2. The Rietveld refinement results of (**a**) LZGO and (**b**) LZGS6%O:0.8%Cr^{3+}

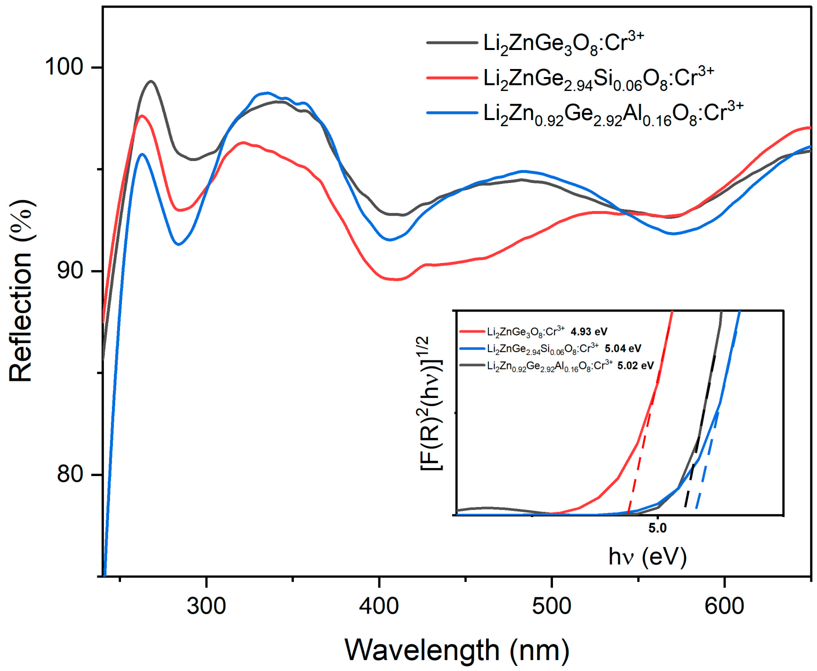

Figure S3. The DRs of LZGA: Cr, LZGS: Cr and LZGO: Cr. Inset: the bandgap width of LZGA: Cr, LZGS: Cr and LZGO: Cr.

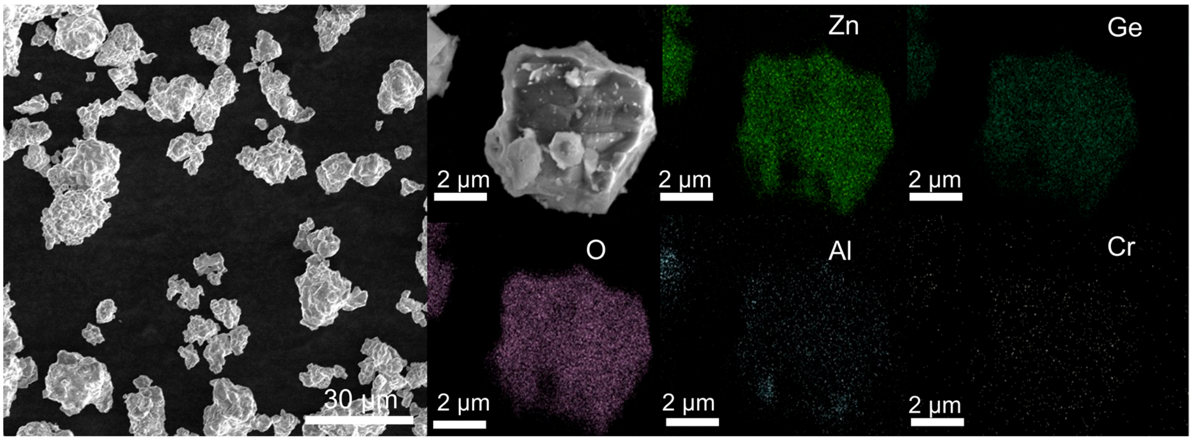

Figure S4. Elemental mapping analysis and SEM of LZGAO:0.8%Cr^{3+}.

Table S1. Refined crystallographic data of LZGS: Cr (y = 0-12%)

**y**	**0%**	**2%**	**6%**	**8%**	**12%**
Crystal System			Cubic		
a(Å)=b(Å)=c(Å)			P4332 (Allows Chirality)		
Cell Voluma ($Å^3$)	550.414	550.300	550.259	550.287	550.317
R_{wp} (%)	12.6	12.2	10.57	13.2	14.5
R_p (%)	9.0	8.69	8.63	9.38	9.96
χ^2	1.311	1.236	1.450	1.419	1.702

Table S2. Refined crystallographic data of LZGA: Cr (y = 0-12%)

**z**	**0%**	**0.5%**	**2%**	**6%**	**10%**
Crystal System			Cubic		
a(Å)=b(Å)=c(Å)			P4332 (Allows Chirality)		
Cell Voluma ($Å^3$)	550.414	550.403	550.327	550.330	550.351
R_{wp} (%)	12.6	11.9	12.0	12.4	12.7
R_p (%)	9.0	8.64	8.73	8.69	9.11
χ^2	1.311	1.286	1.297	1.381	1.455
